# Transfer RNA-Derived Small RNAs: Novel Regulators and Biomarkers of Cancers

**DOI:** 10.3389/fonc.2022.843598

**Published:** 2022-04-28

**Authors:** Bi-Fei Fu, Chao-Yang Xu

**Affiliations:** Department of Breast and Thyroid Surgery, Affiliated Jinhua Hospital, Zhejiang University School of Medicine, Jinhua, China

**Keywords:** tsRNAs, human carcinoma, biomarker, carcinogenesis, diagnosis

## Abstract

Transfer RNA-derived small RNAs (tsRNAs) are conventional non-coding RNAs (ncRNAs) with a length between18 and 40 nucleotides (nt) playing a crucial role in treating various human diseases including tumours. Nowadays, with the use of high-throughput sequencing technologies, it has been proven that certain tsRNAs are dysregulated in multiple tumour tissues as well as in the blood serum of cancer patients. Meanwhile, data retrieved from the literature show that tsRNAs are correlated with the regulation of the hallmarks of cancer, modification of tumour microenvironment, and modulation of drug resistance. On the other side, the emerging role of tsRNAs as biomarkers for cancer diagnosis and prognosis is promising. In this review, we focus on the specific characteristics and biological functions of tsRNAs with a focus on their impact on various tumours and discuss the possibility of tsRNAs as novel potential biomarkers for cancer diagnosis and prognosis.

## Introduction

Non-coding RNAs (ncRNAs) that exist widely in cells are not translated into proteins and can be roughly divided into two categories: small ncRNAs (sncRNAs) and long ncRNAs (lncRNAs) (1). Recent sequencing data reveal that ncRNA transcripts are four times than protein-coding RNA transcripts in human cells (2, 3). However, the majority of ncRNAs and their functions in mammals are underappreciated (4). LncRNAs are longer than 200 nt and include the most nuclear ncRNAs (5). SncRNAs are up to 200 nt in length and mainly include small interfering RNAs (siRNAs), microRNAs (miRNAs), PIWI-interacting RNAs (piRNAs), and small nucleolar RNAs (snoRNAs) (6–8). These sncRNAs have been extensively studied in the past two decades and exert complex roles in biological processes, such as inhibition of translation, metabolic modulation and others (9–11). In this review, we will focus on an emerging functional sncRNA called transfer RNA-derived small RNAs (tsRNAs).

Transfer RNAs (tRNAs) are a group of classic sncRNAs, which transport amino acids to messenger RNAs (mRNAs) during the process of translation (12). The typical two-dimensional configuration of mature tRNAs has a stem-loop structure. As it has been reported in previous studies, from the 5’ to 3’ end the stem-loop constructions compose the acceptor stem, the D-loop, the anti-codon loop, the variable loop and the TψC loop (13). Recent data have unveiled the role of tRNAs’ posttranscriptional modifications to dysregulation of certain genes resulting in the development of different human diseases (14, 15).

As a novel group of sncRNAs derived from tRNAs, transfer RNA-derived small RNAs (tsRNAs) are produced by specific nucleases (16). They cut specific sites of the precursor or mature tRNAs, when found under stress, infection, tumorigenesis, and other specific conditions, which are barely connected with parental tRNA abundance (17–20). Recently, multiple tsRNAs have been discovered in various tumour cells (21). Meanwhile, emerging evidence has suggested that tsRNAs play a critical role in regulating cancer hallmarks, modifying tumour microenvironment, and modulating drug resistance by involving multiple biological processes, such as inhibition of mRNA translation, promotion of ribosome biogenesis, and regulation of epigenetic processes (22–26). Moreover, these findings highlight that tsRNAs can potentially be considered as biomarkers for cancer diagnosis and prognosis (27–29).

Herein, we provide a comprehensive summary of the tsRNAs’ characteristics. Furthermore, we discuss the dysregulations and functions of tsRNAs in numerous cancers and explore the possibility of tsRNAs as diagnostic and prognostic biomarkers for cancers.

## Biogenesis and Characteristics of tsRNAs

**Figure 1:**
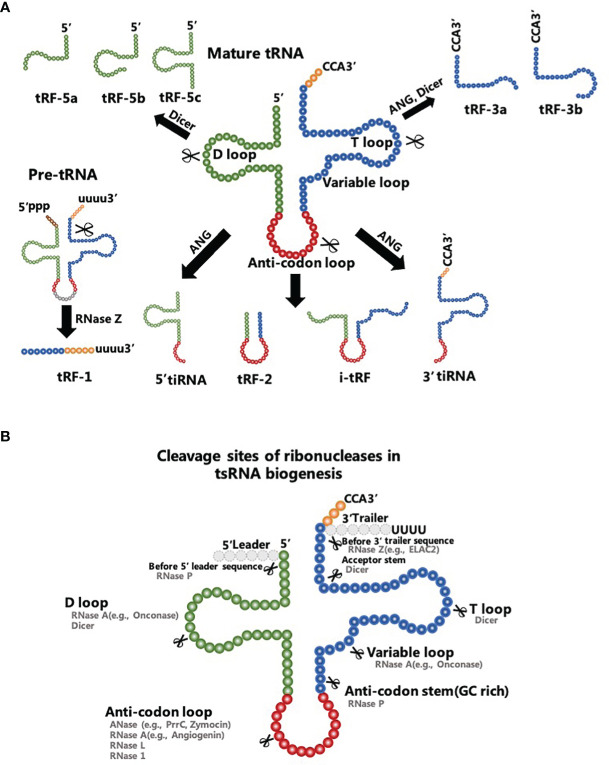
Biogenesis and characteristics of tsRNAs. **(A)** tRF-1s are products of the RNase Z clipping off the 3’-end of precursor tRNAs. tRF-5s are generated from the 5’-tail of mature tRNAs to the D-loop or the sequence between the D-loop and the anti-codon loop of a mature tRNA. tRF-3s are derived from the TψC loop to the 3’ end of mature tRNAs. tRF-2s comprise the sequence straddling the anti-codon loop of mature tRNAs. i-tRFs comprise the anti-codon loop of mature tRNAs and sections of the D-loop and T-loop. **(B)** Ribonucleases in tsRNA biogenesis include Dicer, RNase P, RNase 1 and others with their known cleavage sites.

tsRNAs are conventional sncRNAs that can be classified into two major subtypes: tRNA-related small RNA fragments (tRFs) with a length of 18-30 nt and tRNA halves with a length of 30-40 nt (**Figure 1A**). tsRNAs were initially classified as degradation debris of tRNAs during biological processes and were closely related to parental tRNA abundance. However, accumulating evidence indicates that tsRNAs are enzyme-digested products of exact nucleases that cut into concrete sites of precursor or mature tRNAs under specific conditions, such as stress, infection, neurodegeneration, and tumorigenesis (30–33). In addition, authors report that tsRNAs have various types of RNA modifications, including methylation modification, modification of the 5’-hydroxyl terminal and 2’, 3’-cyclic phosphorylation modification (34). These modifications have been proposed to depend on their precursor tRNAs and the type of enzymatic processing (35). Thus tsRNAs are not considered random tRNA degradation byproducts, but a cluster of functional molecules with high stability and inherent conservation (35, 36). Interestingly, data reveal the pivotal role of all these types of regulatory tsRNAs in post-transcriptional regulation in cancer cells (37, 38).

Five classes of tRFs are known and they are tRF-1s, tRF-2s, tRF-3s, tRF-5s, and i-tRFs (39). Besides the fact that the tRF-1s are products of the ribonuclease Z (RNase Z) clipping off the 3’-end of precursor tRNAs (40), other tRFs have proven to be enzyme-digested products of mature tRNAs (41). Dicer, angiogenin (ANG), and other particular ribonucleases participate in the digesting processes of these mature tRNAs (**Figure 1B**). A tRF-5s is generated from the 5’-tail of a mature tRNA to the D-loop (tRF-5a) or the sequence between the D-loop and the anti-codon loop (tRF-5b and tRF-5c) of a mature tRNA. tRF-5s are typically produced by Dicer and ANG cleavage into the different termination site between 5’ end and the anti-codon loop. tRF-3s that mainly contain tRF-3a and tRF-3b are derived from the TψC loop to the 3’ end of mature tRNAs. Dicer is the main ribonuclease cutting into the TψC loop to produce tRF-3s. In that sense, tRF-2s comprise the sequence straddling the anti-codon loop of mature tRNAs with variable lengths. Alternatively, i-tRFs comprise the anti-codon loop of mature tRNAs and sections of the D-loop and T-loop. The specific mechanism of tRF-2s/i-tRFs production remains unclear (42).

tRNA halves, as the name suggests, are derived from the 5′ end of mature tRNAs to the terminus of the anti-codon loop (5′-tRNA halves) or start at the anti-codon loop and proceed to the 3’ end of mature tRNAs (3′-tRNA halves). Findings retrieved from the literature indicate that these tsRNAs are mainly involved in hypoxic conditions, nutritional deficiency, heat shock, and other conditions of stress (43). Therefore, tRNA halves are universally called tRNA-derived stress-induced RNAs (tiRNAs) that act as effectors of cellular stress responses (44). tiRNAs are known to be produced by ANG which knockdown considerably reduces the level of tiRNAs in human U2OS cells (45). In a recent study, Su et al. found that production of certain tiRNAs was dependent on RNase L cleavage and hence ANG was not the only ribonuclease to produce tiRNAs (46). Interestingly, the other type of tRNA halves known as the sex hormone-dependent tRNA-derived RNAs (SHOT-RNAs) are not induced by various stress stimuli, but they are highly expressed in hormone receptor-positive breast and prostate cancer cells (47). Therefore, SHOT-RNAs represent a separated category of tRNA halves with distinct specificity of expression (48).

tsRNA biogenesis is regulated by tRNA modifications. It has been reported that two (cytosine-5) RNA methyltransferases, DNMT2 and NSUN2, add 5-methylcytosine (m^5^C) modification to particular tRNAs. Thus they protect tRNAs from cleavage into tsRNAs in mice (49). The tRNA methyltransferase 10 homolog A (TRMT10A) was also found to mediate N1-methylguanine (m^1^G) modification to several tRNAs and decrease tsRNA’s production (50). In addition, the queuine tRNA-ribosylthansferase catalytic subunit 1 (QTRT1)-dependent addition of queuosine (Q) modification to several tRNAs increases tRNA stability in HEK293T cells (51). Moreover, 2’-O-methylation modification of the C34 residue in the tRNA^Met^ can inhibit tRNA degradation by ANG and decrease tsRNA production (52). Except for preventing tsRNA biogenesis, some tRNA modifications have promoted tRNA cleavage into tsRNAs (35). For example, pseudouridylate synthase 7 homolog (PUS7)-dependent addition of pseudouridine (Ψ) modification to several tRNAs promotes tsRNA biogenesis in stem cells (53). A recent study demonstrated that the knockout of ALKBH1 or ALKBH3 genes increases N1-methyladenine (m^1^A) modification in several tRNAs and lowers the abundance of tsRNAs in human 293T cells (54). In another study, the 5-methoxycarbonylmethyl-2-thiouridine (mcm^5^s^2^U) modification at position 34 (wobble position) was found to promote efficient cleavage of substrate tRNAs into yeast tsRNAs (55). In this regard, it is of great importance to mention that tRNA modification not only correlates to tsRNA biogenesis but is also associated with changes in tsRNA functions. The last occurs due to the abovementioned modifications, which have posed challenges for tsRNA library preparation and the conduction of studies concerning the mechanism of tsRNAs.

To date, the next-generation sequencing data allowed a deeper analysis of the obtained evidence, researchers have made remarkable progress in terms of the biogenesis and classification of tsRNAs. Meanwhile, online databases like tsRBase and OncotRF providing validated tsRNAs are emerging (56, 57) (**Table 1**). The last is a result of the fact that important biological processes have been demonstrated to be strongly correlated with tsRNAs, which has drawn broad attention, especially in cancer studies.

**Table 1:** tsRNA databases.

Database	Description	URL link
tRFdb	A relational database of tRFs	http://genome.bioch.virginia.edu/trfdb/
MINTbase	A database for interaction of mitochondrial and tRFs	http://cm.jefferson.edu/MINTbase/
tRFexplorer	A database shows tRFs expression profile in each TCGA tumor type	https://trfexplorer.cloud/
tRF2Cancer	A database identifies tRFs from sequencing datasets in various cancers	http://rna.sysu.edu.cn/tRFfinder/
OncotRF	A database provides the comprehensive tRF information related to various cancers	http://bioinformatics.zju.edu.cn/OncotRF
tsRBase	A comprehensive database for tsRNA expression and function	http://www.tsrbase.org/search.php

## Biological Processes Correlated With tsRNAs

**Figure 2:**
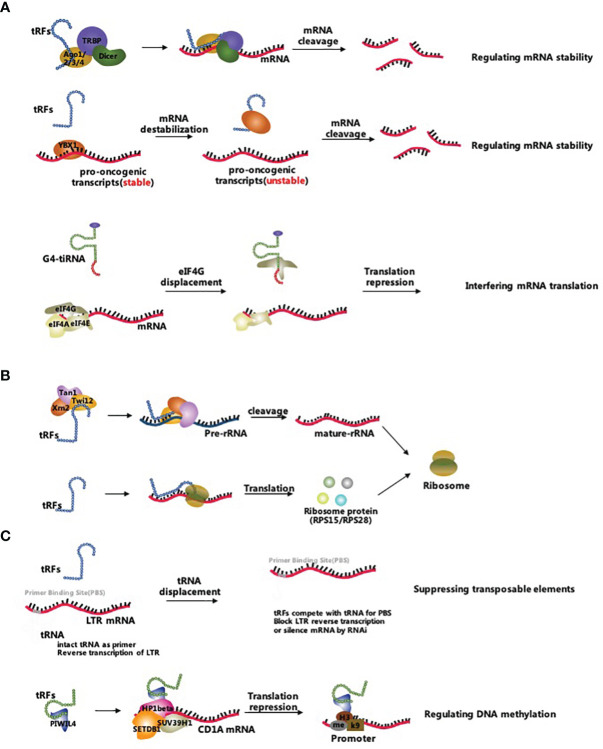
Biological functions of tsRNAs. **(A)** tsRNAs inhibit mRNA translation by reducing mRNA stability and interfering mRNA translation; **(B)** tsRNAs promote ribosome biogenesis by regulating rRNAs and ribosomal proteins; **(C)** tsRNAs regulate epigenetic processes by suppressing transposable elements and targeting the DNA methylation.

The biological processes correlated with tsRNAs involve multiple pathways such as inhibition of mRNA translation, promotion of ribosome biogenesis, and regulation of epigenetic processes (17). **Figure 2** comprehensively summarizes the three main tsRNA-associated molecular mechanisms (**Figure 2**).

### Inhibition of mRNA Translation

tsRNAs are thought to inhibit mRNA translation by either regulating mRNA stability or interfering with translation initiation and elongation (19). Unlike other sncRNAs, tsRNAs regulate mRNA stability by either canonical miRNA pathway, binding to Argonaute 2 (Ago2) protein or non-canonical miRNA pathway, incorporating into other Argonaute (Ago) family proteins (58, 59). For example, CU1276 was considered as a type of tRFs in human germinal centre B cells and was a DICER1-mediated cleavage production of tRNA-Gly. This specific tRFs repressed replication protein A1 (RPA1) protein by complementarily targeting the 3’UTR of its mRNA, thus suppressing proliferation. More specifically, CU1276 incorporated into silencing complexes with each of the four Argonautes *via* analysis of coprecipitated RNA using an Ago-reactive antibody in human kidney 293 cells (60). A study conducted on colorectal cancer cells showcased that a tRF derived from tRNA^Leu^ functions as miRNAs and inhibits the Notch pathway by interacting with the 3’UTR of Notch ligand jagged 2 (JAG2) mRNA, and suppresses cancer stem-like cells in colorectal cancer progress (61). Li et al. found that some ANG-dependent tRF-3s cleaved the target mRNA by guiding the Ago2 protein, which was similar to the miRNA induced silencing complex (62). Concerning the miRNA-like gene silencing pathway, Haussecker et al. found that some tRFs regulated mRNA stability *via* binding to Ago proteins except for the Ago2 protein forming in this way tRF-induced silencing complexes (63). Subsequent reports indicated that these tRF-induced silencing complexes probably targeted the coding regions, 3’UTRs and 5’UTRs of mature mRNAs (64–66). Furthermore, tRFs mainly derived from tRNA-Glu, tRNA-Asp, tRNA-Gly, and tRNA-Tyr were demonstrated to competitively bind to Y-box-binding protein 1 (YBX1), which enabled the displacement of multiple oncogenic transcripts from the YBX1 protein in breast cancer cells. Subsequently, this led to a reduction in the stability of oncogenic transcripts and inhibited the progress of breast cancer cells (67).

A considerable body of evidence has shown that tsRNAs inhibited translation by interfering with translation initiation and elongation. Specifically, Lyons et al. identified that G-quadruplex (G4) structures containing tiRNAs (G4-tiRNAs) displaced eIF4G, impaired the assembly of 40S ribosomal subunit and ultimately inhibited the translation initiation (68, 69). Moreover, Gebetsberger et al. demonstrated that a tRF-5s derived from tRNA-Val acted as a translation elongation brake for the polysome assembly in *Haloferax volcanii*, which bound the small ribosomal subunit and repressed the consequent protein biosynthesis (70).

Briefly, the piled-up evidence proposes that some tsRNAs probably weaken protein biosynthesis by inhibiting translation processes. Further efforts are needed to explore the regulatory networks between mRNA targets and tsRNAs.

### Promotion of the Ribosome Biogenesis

Recent studies proved that some tsRNAs promoted ribosome biogenesis *via* the regulation of ribosomal RNAs (rRNAs) and ribosomal proteins (22). An example of the last assertion is the tRF-3s in *Protozoa* that was shown to recruit the *Tetrahymena* Piwi 12 (Twi12), exoribonuclease 2 (Xrn2) and Twi-associated novel 1 (Tan1) proteins to compose the pre-rRNA splicing complex Twi12/Xrn2/Tan1 (TXT), which processed the precursor rRNAs to mature rRNAs (71, 72). However, whether tRFs promoted rRNA production in higher-level classified living organisms is still unknown. Additionally, a group of tRF-3s derived from tRNA^Leu^ was shown to bind RPS28 or RPS15 mRNAs to promote ribosomal protein translation (22, 73). Yet, the available evidence is insufficient to indicate the extent of the relation of tiRNAs to ribosome biogenesis. In conclusion, the regulatory pathways of tsRNAs role in ribosome biogenesis are the focus for future studies.

### Regulation of Epigenetic Processes

tsRNAs have gradually emerged as a novel epigenetic factor. On one hand, tsRNAs act as epigenetic regulators that maintain the genome’s stability by targeting and suppressing transposable elements (23). Studies have shown that some tRF-3s silenced the LTR-retrotransposons by blocking reverse transcription and by post-transcriptional silencing (74, 75). Furthermore, abundant tiRNAs in the mature sperm altered the transcriptome of mouse embryos without changing the DNA methylation status. The observed phenomenon indicated that tiRNAs acted as epigenetic factors in the mature sperm but also affected the next-generation phenotyping (76–78). On the other hand, tsRNAs regulated the epigenetic processes *via* targeting DNA methylation and histone modifications (79). Data have revealed that tRF-5s derived from tRNA-Glu was combined with Piwi-like protein 4 (PIWIL4) thus recruiting the SET domain bifurcated histone lysine methyltransferase 1 (SETDB1), SUV39H1 histone lysine methyltransferase (SUV39H1), and heterochromatin protein 1β (HP1β) proteins leading to the methylation of the promoter region and inhibition of CD1A expression in monocytes (80, 81). These findings suggest that a new chapter has been unfolded regarding the tsRNA-related epigenetic regulations, but yet there is a shortage of knowledge concerning the precise functions of tsRNAs in the epigenetic control, which eventually will be determined on a case-by-case basis.

### The Role of tsRNAs in Cancer Development and Promotion

The functions of tsRNAs have drawn broad attention across the scientific world concerning cancers. So far, numerous studies have demonstrated that tsRNAs have pivotal functions in regulating proliferation, apoptosis, and migration of cancer cells, modifying tumour microenvironment, and modulating cancer drug resistance (29). Moreover, recent clinical research has revealed that tsRNAs were commonly detected in the serum samples from tumour patients (82). Therefore, tsRNAs have great potential to serve as novel biomarkers for various types of cancers, such as breast, lung, colorectal, ovarian and other types (**Table 2**).

**Table 2:** Summary of cancer-associated tsRNAs.

Cancer	tsRNAs	Effect	Mechanism	Clinical Value	References
Breast cancer	tRF^Glu-YTC^ tRF^Asp-GTC^ tRF^Gly-TCC^	Tumor suppressor	Displace oncogenic transcripts from YBX1 and then suppress cell proliferation	Need to be further studied	(67)
	tiRNA^Asp-GUC^ tiRNA^His-GUG^ tiRNA^Lys-CUU^	Tumor promoter	Involvement in cell proliferation	Potential biomarker for ER^+^ patient	(47)
	ts-112	Tumor promoter	Target RUNX1 and promote tumor-related activities	Need to be further studied	(83)
	tRF^Lys-CTT-010^	Tumor promoter	Interact with G6PC and then promote cell proliferation	Need to be further studied	(84)
	tRF-Arg-CCT-017, tRF-Gly-CCC001, tiRNA-Phe-GAA-003	Unknown	Unknown	Diagnostic and prognosis biomarker	(85)
	tRF-31-87R8WP9I1EWJ0	Unknown	Unknown	Diagnostic and prognosis biomarker	(86)
	tDR‐000620	Unknown	Unknown	Diagnostic biomarker	(87)
	tRF3E	Tumor suppressor	Interact with nucleolin and repress the translation of P 53	Diagnostic biomarker	(88)
	tRF-30-JZOYJE22RR33 ,tRF-27-ZDXPHO53KSN	Unknown	Unknown	Prognostic biomarker	(89)
Gastric cancer	tiRNA-5034-GluTTC-2	Unknown	Unknown	Diagnostic biomarker	(90)
	tRF-Glu-TTC-027	Tumor suppressor	Regulate progression via MAPK pathway	Potential target for clinical therapy	(91)
	tRF-19-3L7L73JD	Tumor suppressor	Inhibit proliferation and Promote apoptosis	Diagnostic biomarker	(92)
	tRF-33-P4R8YP9LON4VDP	Tumor suppressor	Inhibit proliferation and migration	Diagnostic biomarker	(93)
	tRF-24-V29K9UV3IU	Tumor suppressor	Inhibit proliferation and migration by regulating the Wnt pathway	Potential target for clinical therapy	(94)
Colorectal cancer	tRF-24-NMEH623K25, tRF-30- XSXMSL73VL4Y, tRF-29- QU7BPN6ISBJO, tRF-27- Q99P9P9NH5N	Tumor promoter	Regulate the progression of colon cancer via cGMP-PKG signaling pathway	Diagnostic biomarker	(95)
	tRF/miR-1280	Tumor suppressor	Suppress stem cell-like cells and metastasis	Potential diagnostic marker	(61)
	tiRNA^His-GTG^	Tumor promoter	Regulate LATS2 and promote progression	Need to be further studied	(26)
	50-tiRNA-Val, 50-tiRNA-Cys, 50-tiRNA-Ala	Tumor promoter	Promote migration and invasion	Potential target for diagnosis	(96)
	tRF-20-M0NK5Y93	Tumor suppressor	Inhibit metastasis by targeting Claudin-1	Need to be further studied	(97)
Prostate cancer	tRF-1001	Tumor promoter	ELAC2 dependent production and promote proliferation	Need to be further studied	(40)
	tiRNA^Asp-GUC^ tiRNA^His-GUG^ tiRNA^Lys-CUU^	Tumor promoter	Involvement in cell proliferation	Potential diagnostic marker	(47)
	tRF-544 tRF-315	Tumor promoter	Target GADD45A and protect cancer cells from apoptosis	Prognostic biomarker for recurrence	(98, 99)
Lung cancer	tRF^Leu-CAG^	Tumor promoter	Regulate AURKA and increase proliferative ability of cancer	Potential diagnostic marker	(100)
	ts-46 ts-47	Tumor suppressor	Interfere with S1P pathway and inhibit cancer progress	Need to be further studied	(17)
	ts-3676 ts-4521	Tumor suppressor	Interact with PIWI proteins and inhibit cancer progression	Need to be further studied	(101)
Hepatocellular cancer	tRNA-ValTAC-3, tRNA-GlyTCC-5, tRNA-ValAAC-5, tRNA-GluCTC-5	Unknown	Unknown	Diagnostic biomarker	(102)
	tRF^Gly^	Tumor promoter	Promote cell migration by targeting NDFIP2	Potential therapeutic target	(103)
Ovarian cancer	tRF-03357	Tumor promoter	Promote proliferation by targeting HMBOX1	Potential diagnostic marker	(104)
	tRF-03358	Unknown	Unknown	Diagnostic biomarker	(105)
Pancreatic cancer	tRF-3-Leu-AAG-1-1, tRF-3-Gln-CTG-1-1, tRF-3-AlaCGC-1-1	Tumor promoter	Target cancer-related pathways	Diagnostic biomarker	(106)
	tRF-Pro-CGG	Tumor suppressor	inhibits the metastasis of pancreatic cancer	Diagnostic biomarker	(85)
Leukemia	i-tRF-Phe^GAA^	Unknown	Unknown	Biomarker for prognosis	(107)
	i-tRF-Gly^GCC^	Unknown	Unknown	Prognostic biomarker	(108)
	i-tRF-Gly^CCC^	Unknown	Unknown	Diagnostic and prognostic biomarker	(109)
	ts-101, ts-53, ts-44	Tumor suppressor	Target TCL1 and suppress progression	Need to be further studied	(110)
	tRF-Leu^AAG/TAG^	Unknown	Unknown	Prognostic biomarker	(111)
Renal cell carcinoma	tRF^Val-AAC^ tiRNA^Leu-CAG^-5 tiRNA^Arg-CCT^-5 tiRNA^Glu-CTC^-5 tiRNA^Lys-TTT^-5	Tumor suppressor	Act in a tumor-suppressive manner	Prognostic biomarker	(112, 113)
Osteosarcoma	tiRNA^Ala^ tiRNA^Cys^	Tumor promoter	Trigger assembly of stress granules	Need to be further studied	(114)
B cell lymphoma	CU1276/tRF-3018	Tumor suppressor	Associate with Ago proteins and suppress proliferation		(60)
Bladder cancer	5’-tRF-Lys-CTT	Unknown	Unknown	Diagnostic and prognosis biomarker	(115)
Papillary thyroid cancer	tRF-39-0VL8K87SIRMM12E2, tRF-38-0VL8K87SIRMM12V tRF-34-YSV4V47Q2WW1J1, tRF27-PIR8YP9LON3	Unknown	Unknown	Diagnostic biomarker	(116)
Oral squamous cell carcinoma	tRF-20-S998LO9	Unknown	Unknown	Prognosis biomarker	(117)

### Dysregulations of tsRNAs in Cancers

The first abnormally expressed tsRNA (tRF-1001) was found in various cancer cells (40) and since then the number of dysregulated tsRNAs have increased. The role of tsRNA in oncology research was revisited and many entered clinical trials. In that sense, Fabris and colleagues reported a significant downregulation of ts-53 and ts-101 in chronic lymphocytic leukaemia (CLL) (110). Similarly, Maute et al. observed that some tRF-3s were also downregulated in germinal centre-derived lymphomas (60). tDR-7816 was distinctly downregulated in breast cancer cells (118, 119). However, in cancer tissues from non-small cell lung cancer (NSCLC), the tRF-Leu-CAG was significantly upregulated, while the serum level of tRF-Leu-CAG was positively correlated to cancer stages (100). Likewise, Papadimitriou et al. reported a significant elevation of the tRF-Lys-CTT in bladder tumors and a positive association with clinical prognosis (115). Interestingly, data demonstrated diverse expression of three different tRF-5s in the testicular germinoma (120). As a result, data suggested that deregulation of tsRNAs turned to be a key factor in the progress of cancers thus introducing the tsRNAs as cancer biomarkers in the medical practice.

### The Role of tsRNAs in the Regulation of Cancer Hallmarks

Cell proliferation, apoptosis, and migration are well-known hallmarks of cancers and determinants of the prognosis of cancer patients. tRF-1001 derived from the pre-tRNA^Ser^ was the first tsRNA found to promote the proliferation of prostate cancer cells (40). Moreover, Lee et al. found that the knockdown of tRF-1001 impaired cell proliferation and resulted in the accumulation of cells in interphase with phenotypes reversed by transfecting cells with the synthetic tRF-1001 oligoribonucleotide. In another study, SHOT-RNAs were significantly upregulated in hormone receptor-positive prostate and breast carcinoma cells, and the proliferation of cancer cells was distinctly decreased when transfecting cancer cells with SHOT-RNA-targeted siRNAs (47). These observations suggested that SHOT-RNAs probably stimulated cell proliferation in hormone receptor-positive prostate and breast carcinoma cells. Regarding ovarian cancer cells, Zhang et al. found that tRF-03357 was upregulated and that this tsRNA promoted the proliferation of ovarian cancer cells *via* downregulation of the Homeobox-containing protein 1 (HMBOX1) transcription factor (104). On the other hand, the expression of HMBOX1 in high-grade serous ovarian cancer cells was significantly lower than that in normal ovarian cells. However, the specific pathway involved in tRF-03357 regulating HMBOX1 and other tRFs that modulated the progression of ovarian cancer required further investigation. Contrary to tRF-03357, a tRF-5s derived from tRNA-Glu has been elucidated to target the breast cancer anti-estrogen resistance protein 3 (BCAR3) and hence to inhibit the proliferation of ovarian carcinoma cells (121). Zhou and colleagues demonstrated that the decrease in the expression of BCAR3 and the increase of tRF suppressed the proliferation of ovarian cancer cells. The authors further confirmed that the same tRF bound directly to the 3’UTR of BCAR3 mRNA, which then resulted in downregulation of the BCAR3 protein. Furthermore, previous studies have suggested that tRF-Leu-CAG affected the proliferation of lung carcinoma cells by targeting the Aurora Kinase A (AURKA) protein, which participated in the control of cell cycle and the regulation of the cell division (100, 122). Even more, a recent study showed that tsRNA-5001a was significantly upregulated in lung adenocarcinoma tissues and the overexpression of tsRNA-5001a significantly promoted cell proliferation (123). Hu et al. discovered that tsRNA-5001a promoted cancer cell proliferation by targeting the growth arrest and DNA damage inducible gamma (GADD45G) and downregulating its expression (123). GADD45G is widely known for its antitumor function (124). However, the relationship between tRFs and other DNA repair genes similar to GADD45G in lung adenocarcinoma patients needs to be further explored. In this regard, Maute et al. demonstrated that CU1276, as one of the representatives of tRF-3s, inhibited the proliferation of B cell lymphoma *via* the RPA1-dependent pathway (60, 125). Additionally, Shen et al. found that levels of tRF-33-P4R8YP9LON4VDP were significantly downregulated in plasma samples of gastric patients and that this tsRNA inhibited the proliferation of gastric carcinoma cells (93).

Cell apoptosis, also known as programmed cell death, is an important hallmark of cancer cells (126). Recently, it has been suggested that tRF-315 targeted the growth arrest and DNA damage inducible alpha (GADD45A) gene known for being a tumour suppressor, which then regulated the cell cycle, and finally protected the prostate cancer cells from apoptosis (99). Furthermore, 5’-tiRNA-His targeted the large tumour suppressor kinase 2 (LATS2) protein to turn off the downstream signalling pathway and finally upregulated the anti-apoptotic-related genes (26). Alternatively, Elbarbary et al. found that 5’-half-tRNA(Glu), working as sgRNA, stimulated tRNA 3’ processing endoribonuclease (tRNase Z) to cleave the target protein phosphatase 1F (PPM1F) mRNA and thus affected the apoptosis of human kidney 293 cells *via* suppressing the expression of the PPM1F protein (127). These data are an indication that various tsRNAs are negatively connected with the apoptosis of cancer cells. On the contrary, in breast carcinoma, 5′-tiRNA-Val was shown to alter the colony formation. Another study confirmed that 5′-tiRNA-Val targeted the Frizzled-3 (FZD3) protein, attenuating the Wnt/β-catenin pathway, and finally promoting the apoptosis of cancer cells (118).

Cell migration is also the main trait of malignant tumours and indicates the degree of cancer progression (128). Retrieved data from clinical trial research link distant metastasis with significant tRFs dysregulations in uveal melanoma (129). Further, Birch et al. found that these dysregulated tRFs affected the retrotransposon activity and probably played a pivotal role in cancer cell migration (130). Even more, a tRF derived from tRNA^Leu^ was determined as an inhibitor of migration by preventing the premetastatic niche (PMN) formation in colorectal cancer cells (131). The endothelial–mesenchymal transition (EMT) is a critical factor for the regulation of the cancer cells’ migration (132). Recently, it has been demonstrated that tRFs derived from tRNA-Gly targeted the EMT-related proteins and regulated the migration of hepatocellular carcinoma (103). Regarding other malignancies, tsRNAs were shown to regulate the migration of cancer cells. Specifically, tRF-3019a was found to promote the migration of gastric cancer cells *via* targeting tumour suppressors, while tRF-17-79MP9PP inhibited the migration of breast cancer cells *via* regulating the thrombospondin 1 (THBS1)-mediated transforming growth factor beta 1 (TGF-β1)/SMAD family member 3 (smad3) signalling pathway (133, 134).

### The Role of tsRNAs in Modifying the Tumour Microenvironment

The tumour microenvironment (TME) consists of tumour cells, cancer stem cells, as well as of tumour stromal cells including endothelial cells, fibroblasts, and immune cells, in addition to non-cellular components of the extracellular matrix (135). In recent years, it has been demonstrated that tsRNAs orchestrated the tumour processes by modifying the TME (136). On the one hand, accumulating evidence has shown that exosomes produced by tumour cells possess multiple tRFs that modify the TME (137). For example, exosomes in oral squamous cell carcinoma (OSCC) were shown to contain multiple tsRNAs that are involved in transforming TME to conditions favourable for cancer progression (25). However, the mechanism through which this favourable environment for OSCC growth and metastasis is promoted by tsRNAs remains unknown and needs further studies. Interestingly, exosomal tsRNAs derived from tRNA-Val, tRNA-Gly, and tRNA-Glu were shown to participate in the modulation of TME in hepatic carcinoma (137). Regardless of the above-discussed, the specific biological functions of exosome-derived tRFs in tumour regulation remain to be further investigated. On the other hand, recent studies have drawn increasing attention to cancer stem cells (CSCs) as important components of the TME, while they are involved in numerous tumour biological processes (138, 139). Meanwhile, data indicated that some tRFs have modified the TME by regulating the CSC functions (140). Huang and colleagues reported that tRFs derived from tRNA^Leu^ were found to suppress CSC functions *via* the inactivation of Notch signaling in colorectal cancer cells (61). Recently it has been found that dysregulation of tRF-mediated translational regulatory circuitry impaired the stem cells growth, which was commonly associated with aggressive characteristics of human myelodysplastic syndromes (53). In summary, TME were modified by tsRNAs, but the underlying molecular mechanisms of tsRNAs that regulated the TME remained yet not fully understood. Hence, our suggestion for future studies is that the focus should be on the elucidation of these mechanisms.

### The Role of tsRNAs in the Modulation of Tumour Drug Resistance

Drug resistance is considered a vital factor determining the efficacy of anti-cancer therapies. Emerging evidence showed that some tsRNAs modulated the drug resistance of multiple tumours (24). For example, Cui et al. found that the upregulated tDR-0009 and tDR-7336 sustained the interleukin-6 reactivity and finally participated in multidrug resistance by activating downstream pathways in triple-negative breast cancer cells (141). Meanwhile, another study showed that tRF-30-JZOYJE22RR33 and tRF-27-ZDXPHO53KSN orchestrated trastuzumab resistance in HER2-positive breast cancer cells (89). Sun et al. studied the expression levels of tRFs in trastuzumab-sensitive and resistant breast cancer cell lines. The authors revealed the effect of tRFs on clinical trastuzumab efficacy using the Cox regression analysis. The obtained results highlight the need for further studies that aim at the accumulation of more data that will clarify the role of tRFs in the pathways that regulate the HER2-positive breast cancer drug resistance toward trastuzumab. In addition, other studies showcased that some downregulated tsRNAs were involved in the chemoresistance of lung cancer cells *via* integrin-linked kinase (ILK) signalling, phosphatase and tensin homolog (PTEN) signalling, and other pathways involved in the regulation of chemo-resistance (17, 142). Therefore, with the accumulating evidence on the role of tsRNA in tumour drug resistance, the clinical efficacy of anti-cancer drugs and the prognosis of cancer patients can progress to a new phase.

### The Role of tsRNAs as Biomarkers for Cancer Diagnosis and Prognosis

Multiple ncRNAs have been regarded as potential biomarkers for cancer diagnosis and prognosis (143, 144). Recent studies have further proposed tsRNAs as novel tumour biomarkers (**Table 2**). On one hand, some tsRNAs have demonstrated dysregulations in cancer tissues and serum samples. Hence, these tsRNAs were demonstrated as potential biomarkers for cancer diagnosis. For example, tRFs derived from tRNA-Met and tRNA-Val have been significantly elevated in the serum of pancreatic ductal adenocarcinoma (PDAC) patients as supposed by Xue and colleagues (82). Moreover, Li et al. found that the expression of tRF-Pro-CGG was significantly downregulated in PDAC than this in normal pancreatic tissues, which was further associated with the TNM stage of patients. Thus, tRF-Pro-CGG has been considered as a biomarker for PDAC diagnosis and therapy (85). Similarly, tRFs derived from tRNA^Leu^ were significantly elevated in NSCLC tissues and serum samples, and therefore they were regarded as a potential biomarker for NSCLC diagnosis (100). In respect of tRFs in lung cancer, such as ts-46, ts-47, ts-3676 and ts-4521, they have been demonstrated as tumour suppressors based on recent reports as to the mechanism (17, 101). However, the clinical value of these tRFs needs to be clarified based on the registered clinical samples. Multiple investigations revealed that some tsRNAs derived from tRNA-Glu, tRNA-Gly, tRNA-Leu, and tRNA-Ser were significantly dysregulated in breast cancer serum samples (145). In another example of the Triple‐negative breast cancer, the expression of tDR‐000620 was strongly correlated with age, node status and local recurrence as proposed by Feng et al. In this study, the multivariate Cox regression demonstrated that the low expression of tDR‐000620 was an adverse predictive factor for recurrence‐free survival (87). Equally important were the findings of Huang et al. who found that tRF-31-U5YKFN8DYDZDD was highly upregulated in serum samples of gastric cancer (GC) patients (146). All these results denoted that circulating tsRNAs could be a potential non-invasive indicator of a cancer diagnosis. On the other hand, these abnormally expressed tsRNAs were established as prognostic models for cancer treatments. For instance, tRF-30-JZOYJE22RR33 and tRF-27-ZDXPHO53KSN were demonstrated to shorten the progression-free survival (PFS) of trastuzumab-resistant breast carcinoma patients (89). Subsequently, data proved that these tRFs were likely to act as biomarkers for the prognosis of trastuzumab-resistant breast carcinoma patients (89). Additionally, another study demonstrated that tRFs derived from tRNA-Lys were associated with a higher risk for progression and poor clinical survival in bladder carcinoma (115). Thus, these data established a prognostic model for bladder cancer based on the expression levels of specific tsRNA. Recently, researchers have also discovered multiple tsRNA signatures in gastric cancer, papillary thyroid cancer, and other malignancies (38, 91, 93). The literature research on this matter highlighted the need for a broader validation of tsRNAs as sensitive biomarkers for cancer diagnosis and prognosis.

## Summary and Perspectives

tsRNAs are a group of conventional small ncRNAs and play complicated roles in most malignancies. Their biological roles include regulation of cancer hallmarks, modification of TME, and modulation of drug resistance. Biofluid screening of miRNAs in clinical practice demonstrated that specific tsRNAs were upregulated in the biofluids of solid and blood malignancies (62, 147). Therefore, the biological functions of multiple tsRNAs in cancers have been widely studied. What is the most important from the aforementioned studies is that these functional tsRNAs potentially serve as novel biomarkers for cancer diagnosis and prognosis. However, for a deeper understanding of the tsRNA-related regulatory networks, further investigation is needed.

Our literature research showed that in the first place, the databases of tsRNAs, especially of tsRNAs in multiple tumours, should be perfected. Recently, biomedical investigations on tsRNAs in multiple cancers have been extensively driven by big data. Thus, OncotRF, tRFdb, and other tsRNA-related databases have been expansively applied in oncological research. However, there is no consensus among the existing databases in terms of the standardized terminology of tsRNAs (57, 148–150). Consequently, the big data analytics for tsRNAs in multiple tumours is yet inefficient. Moreover, current studies have shown that tsRNAs are dysregulated in neoplasm tissues and serum samples of tumour patients. However, our knowledge concerning the upstream regulatory mechanism causing the abnormal expression of tsRNAs and the biological functions of tsRNAs in multiple tumours remains at a relatively superficial level. It is unknown whether the tsRNA dysregulation triggers the progress of tumours. Therefore, it is compulsory to identify the concrete molecular mechanisms of tsRNAs in regulating multiple tumours. Lately, enormous efforts were invested in the exploration of the mechanisms of tsRNAs in cancer cells. To advance clinical application of tsRNAs, future studies should focus on collecting patient samples with warranty of safety and efficacy, such as body fluids and tumour tissues. As it has already been reported, both tsRNAs and their precursor tRNAs contain various modifications (35, 36, 151). Interestingly, the modification status of tRNA can change the endonuclease activity and thus affect the tRNA cleavage process. However, the determination of exactly which tRNA modification affects tsRNA production in cancer progression as well as the potential relationship of tsRNA modification with their functions in tumours needs further investigation. Therefore, it is necessary to establish the localization and detection techniques for examining multiple RNA modifications of tsRNAs in various tumours. Based on detection techniques, the potential future research would focus on investigating upstream regulatory factors of tRNA modifications and endonuclease activity in tumours. Finally, diverse chemotherapies encounter drug resistance, which leads to the necessity of more targeted treatments. Moreover, the advent of RNA therapies bodes for the development of therapeutic approaches involving small RNAs. Thus, some tsRNAs, which are biologically correlated with tumour initiation and progression, are expected to become biomarkers for cancer diagnosis and prognosis. It is worth mentioning that tsRNAs can be encapsulated in exosomes and liquid biopsies based on exosomes can be a potential approach for molecular diagnosis in cancers. For example, some tRFs delivered *via* plasma exosomes served as a novel diagnostic biomarker in the liver cancer (102). These tRFs allow us to understand the pathological conditions of cancer patients by detecting specific tsRNAs encapsulated in exosomes. Moreover, engineered exosomes can load therapeutic miRNAs and anti-tumour drugs (152). Thus, therapeutic tsRNAs are likely to be another potential anti-tumour molecule loaded in engineered exosomes.

Given the abovementioned challenges, most tsRNA-related research on tumours has yet to be profound. Therefore, with the application of novel sequencing techniques and bioinformatics methods, such as photoactivatable-ribonucleoside-enhanced crosslinking and immunoprecipitation (PAR-CLIP) and cross-linking ligation and sequencing of hybrids (CLASH), more in-depth studies of tsRNAs in multiple tumours can provide a brand new insight into tumour cell biology for the establishment of solid foundations for further clinical applications of tsRNAs.

## Author Contributions

C-YX provided direction and guidance throughout the preparation of this manuscript. B-FF wrote and edited the manuscript. B-FF generated the figures and made significant revisions to the manuscript. All authors have read and approved the final version of the manuscript.

## Funding

This study was supported by Jinhua central hospital basic research projects of Zhejiang China(Grant No: JY2021-6-05) and Zhejiang non-profit technology applied research projects of China (Grant No: LGF20H160017).

## Conflict of Interest

The authors declare that the research was conducted in the absence of any commercial or financial relationships that could be construed as a potential conflict of interest.

## Publisher’s Note

All claims expressed in this article are solely those of the authors and do not necessarily represent those of their affiliated organizations, or those of the publisher, the editors and the reviewers. Any product that may be evaluated in this article, or claim that may be made by its manufacturer, is not guaranteed or endorsed by the publisher.
